# Smile In^TM^ Totems in Radiotherapy: Patients’ Satisfaction with Limited Equipment and COVID-19

**DOI:** 10.3390/healthcare10081533

**Published:** 2022-08-13

**Authors:** Marzia Borgia, Fiorella Cristina Di Guglielmo, Marco Lucarelli, Rosario Bonelli, Lucrezia Gasparini, Angelo Di Pilla, Lucia Anna Ursini, Maria Taraborrelli, Annamaria Vinciguerra, Antonietta Augurio, Monica Di Tommaso, Marianna Trignani, Marianna Nuzzo, Consuelo Rosa, Giuditta Chiloiro, Stephanie Sartori, Lucia Ferrari, Roberta Marchione, Fabio Adalgiso D’Orazio, Paola Di Renzo, Giustino Orlando, Domenico Genovesi, Luciana Caravatta

**Affiliations:** 1 Radiation Oncology Unit, SS. Annunziata Hospital, “G. D’Annunzio” University of Chieti, Via dei Vestini, 66100 Chieti, Italy; 2 Department of Neuroscience, Imaging and Clinical Sciences, “G. D’Annunzio” University of Chieti, Via deiVestini, 66100 Chieti, Italy; 3 UOC Radioterapia Oncologica, Dipartimentodi Diagnostica per Immagini, Radioterapia Oncologica ed Ematologia, Fondazione Policlinico Universitario “A. Gemelli” IRCCS—Rome, 00168 Rome, Italy; 4 Associazione E.T.S “IL Tratturo una Strada per la Vita”, Via dei Vestini, 66100 Chieti, Italy; 5 Department of Pharmacy, G. D’Annunzio University, Via deiVestini, 66100 Chieti, Italy

**Keywords:** quality of care, radiotherapy, PREMs, technology assessment, COVID-19

## Abstract

Background: We report a mono-institutional experience regarding patient-perceived quality regarding the Chieti Radiotherapy Department, through RAMSI (Radiotherapy Amica Mia—SmileIN^TM(SI)^—My Friend Radiotherapy^SI^) project, in critical scenarios of limited equipment and COVID-19. Material and methods: Patient-reported experience measures (PREMs) were assessed as follows: Patient-centric welcome perception (PCWP), Comfort, Professional skills and Punctuality. Patients could give anonymous feedback using HappyOrNot technology through four totems located in strategic areas within the center. An internal benchmark was obtained using the feedback received after a preliminary observation period. The SI Experience Index was collected, analyzed and compared. Weekly and monthly reports were generated. Results: From February 2019 to February 2022, 8924 patients accessed the department; 17,464 daily treatments were recorded and 5830 points of feedback were collected: 896, 1267, 1125 and 2542 for PCWP, Comfort, Professional skills and Punctuality, respectively. A LINAC decommissioning period was analyzed, with decreases in the SI-Index score and Smile-IN approved percentage and an improvement after this period. Additionally, the COVID-19 pandemic was analyzed with a mild evaluations decrease for PREM’s Welcome, Comfort and Punctuality (Δ-value: −9%, −3% and −4%, respectively), while Professional skills were always optimal. Conclusion: The RAMSI project was effective for assessing treatment quality perception, allowing for improving clinical procedures with corrective actions. The RAMSI project is ongoing.

## 1. Introduction

The incidence of cancer is increasing and it has become a leading cause of morbidity and mortality worldwide. It is estimated that, by 2025, the number of patients diagnosed with cancer in Europe annually will reach over 4.5 million [1]. Treatment options, including surgery, radiotherapy, chemotherapy, immunotherapy, and other types of innovative treatments, continue to require extensive resources to meet patient needs [2,3]. Radiotherapy represents an integral part of a multidisciplinary cancer program since around 50% of patients needs radiation treatment [4]. This translates into a 16% increase in radiotherapy needs by 2025. Moreover, to date, fewer than three out of four cancer patients with an indication for radiation therapy receive it, with significant disparities among European countries and therefore also in the Italian regions.

Currently in Abruzzo, a region of central Italy with a population of 1,312,000 people, 8383 patients had a cancer diagnosis in 2016, and 7 linear accelerators (LINACs) (2 of which were obsolete and used only for part time shifts) and 2 brachytherapy units were available [5].

Since October 2020, the radiation oncology department of Chieti, in the Abruzzo region, has been characterized by the clinical use of a single linear accelerator (LINAC) due to the obsolescence of second LINACs in equipment, with an increase in waiting times for treatment and an uncomfortable mobility to other centers outside the region for patients.

Therefore, according to Europe estimates, the investments planned in Abruzzo region for radiotherapy will be based on 5000 patients who will need radiotherapy treatment, with an estimated need of 12 LINACs in 2025 [5].

Meanwhile, both the obsolescence and poor availability of resources with a reduced possibility of responding to the need of radiotherapy could have negative impacts on the clinical outcomes and perceptions of the radiation treatment quality and timeliness in terms of possible discontinuities of radiation courses and waiting lists.

Furthermore, COVID-19 produced an important disruption to health care, with a reorganization of hospital activities and disruptions of all planned medical and surgical activities. Among these, oncological and radiotherapy treatments were at risk of being disrupted, even though all patients with cancer were designated as requiring non-deferrable therapies for life-saving care [6,7]. During the COVID-19 pandemic, the radiation oncology department of Chieti adopted an internal protocol in order to ensure radiotherapy (RT) activities, with the exception of clinical follow-up visits according to behavioral guidelines defined both by the hospital management and by experiences shared with other Italian radiotherapy centers [5,6,7,8,9,10,11].

Patients’ satisfaction and their perspectives on the quality of care have become important dimensions for monitoring health care performance. In2005, EORTC developed a 32-item satisfaction with care questionnaire to measure patients’ appraisals of hospital doctors and nurses, as well as aspects of care organization and services (EORTC IN-PATSAT32) [12]. Subsequently, patient-reported experience measures (PREMs) have proven to be an effective tool for measuring the quality of care from the patient’s point of view [13].

Moreover, patients undergoing radiotherapy may suffer discomfort not only because of the clinical conditions but also from daily access to perform treatments [14,15]. Therefore, ensuring professional care and making the patient feel welcomed are desirable.

For these reasons, activating a customer satisfaction tool for patients in a radiation oncology department can allow for evaluating the quality of daily work performed and how to improve its processes [16,17].

In this scenario, the radiation oncology center of Chieti has joined with the RAMSI (Radiotherapy Amica Mia—SmileIN^TM (SI)^—My Friend Radiotherapy SI) project, carried out to allow for the collection and analysis of patient feedback in the form of real-time self-reported experience [16].

We report a three-year mono-institutional experience regarding the quality assessment of radiotherapy performance as perceived by patients mainly in critical scenarios of limited technological equipment and the COVID-19 pandemic.

## 2. Materials and Methods

The RAMSI technology, including four totems with four push buttons using the HappyOrNot technology (RetailIN, Cesano Maderno (MB), Italy-https://smilein.it, access on 2 June 2022), were introduced in the Chieti RT department beginning in February 2019.The total number of feedbacks obtained progressively until February 2022 were analyzed. The data were collected using the HappyOrNot technology: four different faces define four assessment points: very positive, positive, negative and very negative. Patient-reported experience measures (PREMs) were divided as follows: patient-centric welcome perception (PCWP), punctuality, professional skills and comfort. Patients could give their feedback anonymously by pushing a smiling button through four totems set to be pushed once and through the patient identification code only.

Every SI totem was positioned in strategic areas of the department in terms of greater flow of patients, as in the reception waiting room, in the clinic waiting rooms, in the treatment waiting rooms and just outside the LINACs, and each totem had been assigned one of its respective questions:

- *“Did you feel welcomed as a person today?”* for PCWP (reception waiting room);
- *“Are the environments comfortable?”* for Comfort (clinic waiting room);
- *“Have they been competent with you today?”* for Professional skills (treatment waiting room);
- *“Was your treatment schedule respected today?”* for Punctuality (outside the LINAC’s).

The numbers of feedback items for each area were analyzed to evaluate the appropriateness of the questions. A periodic check was carried out to assess the trends in the answers. Data reports were periodically prepared for evaluating patient responses.

Patient ratings were collected in weekly and monthly data reports called a “RAMSI Index” and sent via email to the dedicated clinicians team. A RAMSI data report with “Smile INdex” (SI Index) and “Smile In Approved” (SI Approved) was performed for each area explored. An internal benchmark, obtained by the feedback analysis for the four areas studied and defined every three months during one year, was used. This value was compared with the results obtained in the examined period to evaluate the evidence of the trend.

The SI Index was defined as an approval indicator. Its value (from 0 to 10) is obtained after calculating the weighted average of all the values collected in the evaluation period.

The SI Approved, calculated with values ranging from 0% to 100%, is defined as the percentage of green smiles (very positive + positive) in relation to the total number of votes.

**Figure 1:**
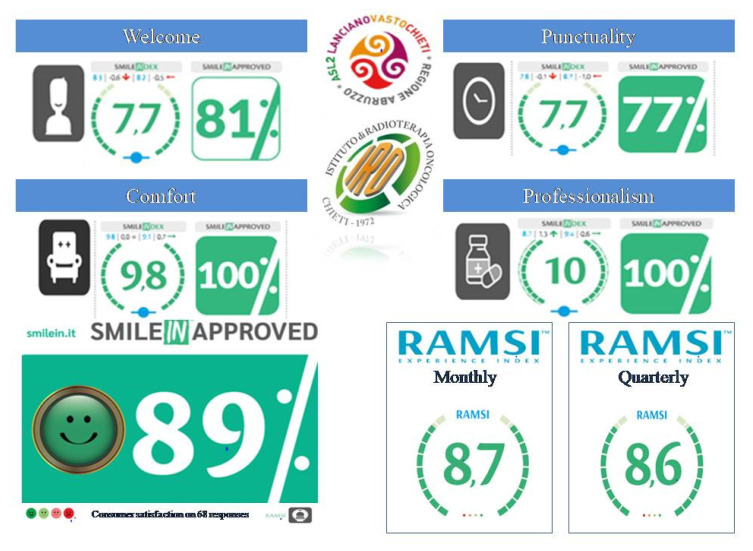
Example of a monthly report of patient-reported experience measures (PREMs), SmileIN-approved and SI Index showed inside the department and available for the patients and clinicians.

The results of the RAMSI system vote are presented inside the department and available for the patients with a monthly report of the different areas investigated by the SmileIN^TM^ totems. The data reported refer to PCWP, punctuality of the visits, therapies, received treatment quality and environmental comfort (Figure 1).

## 3. Results

Since February 2019 to February 2022, a total number of 8924 patients had access to first clinical evaluation and post-treatment follow-up visits, and 17,464 daily treatments were recorded in the radiotherapy department of Chieti.

Overall, 5830 feedback items were collected. The system generates weekly and monthly reports with service satisfaction rates and trends according to time slots and divided into each topic. The report included an SI and SI Approved Indexes with the correspondent internal benchmark value for each question (Table 1).

“Welcome” (PCWP): an SI totem placed in the reception waiting room of the radiotherapy department and asking the question “Did you feel welcomed as a person today?” A total of 896 responses were recorded, with an 86% positive response rate (775 good and very good).

“Comfort”: a SI totem placed in the reception waiting room and asking the question “Are the environments comfortable?” A total of 1267 responses were recorded, with a 92% positive response rate (1163 good and very good).

“Professional skills”: an SI totem located in the treatment waiting room and asking the question “Have they been competent with you today?” A total of 1125 responses were recorded with a 92% positive response rate (1030 positive responses).

“Punctuality”: an SI totem located just outside the LINACs and asking the question “Was your treatment schedule respected today?” A total of 2542 responses were recorded with a 78% positive response rate (1075 positive responses).

An analysis of the hourly and daily patterns of SI was conducted. The peaks of greatest significance were the service opening time for the best score and the lunchtime time for the worst SI related to the punctuality of treatment schedule time.

The minimum feedback by day was on Mondays. This result could be related to the specific activities performed on Mondays, usually dedicated to the clinical evaluation and simulation of palliative treatments.

In the Chieti RT department, one high-tech and one obsolete LINAC were operative during the observational period until November 2020. In this time frame, the obsolete LINAC presented an increasing number of technical failures, leading to several treatment disruptions, delayed treatment starts and the lengthening of waiting lists. At the end of October 2020, the obsolete LINAC has been decommissioned, leaving only one LINAC for clinical use.

An analysis of data was then reported considering the period before and after LINAC decommissioning to evaluate the impact of this discomfort on patients’ perceptions (Table 2). Reductions in the SI index and in the percentages of Smile-in approved were noted before the LINAC decommissioning, probably reflecting patients’ treatment disruptions and delays in treatment start, following improvements in SI and Smile-in approved from November 2020.

An analysis of the COVID-19 period was also performed. In particular, the period from March 9 to 4 May 2019 was examined, with the same time range during the full lockdown phase I of the COVID-19 emergency, from 9 March to 4 May 2020 (Table 3). No changes were observed in the numbers of patients treated. A mild decrease in evaluations was observed, in particular regarding PREM’s welcome, comfort and punctuality (SMILE-IN Approved Δ-value: −9%,−3% and −4% respectively), possibly related to the discomfort that patients experienced related to pandemic issues in health environments. On the other hand, professional skills were less affected by the epidemiological condition (Smile Index Δ-value −0.1 and SMILE-IN Approved Δ-value −1%).

## 4. Discussion

Patients’ satisfaction assessment is recognized as an important tool for evaluating the needs of patients and identifying the areas for improvement in a healthcare organization [14]. This perception may vary according to socioeconomic aspects, healthcare system resources and related patient expectations [17]and can be carried out using different tools even in low-income countries with limited technological models such as the EORTC (EORTC IN-PATSAT32) care questionnaire to measure patients appraisal, care organization and services [12]. Moreover, cancer patients’ concerns regarding the long waiting times for radiation treatment and LINAC disruptions could have negative impacts on quality of care and on patient perceptions of treatment effectiveness. Actions aimed at improving the quality of care are important for their impacts on both patient satisfaction and service organization.

Based on these considerations, the radiotherapy department of Chieti joined the multicentric RAMSI project that put the patient at the center of the therapeutic process to maintain their quality of life (QoL), to evaluate quality of daily work performed and how to improve it.

This project involved other seven radiotherapy centers from 6 Italian regions: “Policlinico S’Orsola-Malpighi”-Bologna, “Spedali Civili”-Brescia, “Policlinico S. Martino”-Genova, “Azienda Ospedaliera Universitaria Policlinico G. Martino”-Messina, “Azienda Ospedaliera”-Perugia, “Policlinico Gemelli”-Roma, “S. Camillo Forlanini”-Roma.

Our data collected among cancer patients appear to be successful and comparable with the results reported by a high-volume and high-tech clinical radiotherapy department with adequate technological equipment as published by Chiloiro and coworkers [16]. In the Chieti experience, high levels of satisfaction have been reported in relation to the professional skills, staff attitudes and department comfort despite a limited number of patients compared with the experience of Chiloiro and coworkers [16]due to the only linear accelerator available for a long time.

Indeed, the highest recorded scores concern professional skills with a Smile Index= 9.0 and a SmileIN approved of 92%. This is probably related to high efforts by the radiotherapy department in the last 20 years to promote a patient-centered model of care as a primary goal of care through the implementation and continuous review of multidisciplinary diagnostic-therapeutic paths for all cancers, the continuous review of clinical and process indicators in the institutional Quality Assurance manuals and the constant clinical research being conducted at Chieti University.

In particular, multidisciplinary tumor boards have been promoted and staffed for the main tumors (breast, prostate, lung, rectal, head and neck and gynecological cancers) [18,19,20,21]. Moreover, simplified and organized clinical models for multidisciplinary and multidimensional evaluations have been implemented such as for frail and elderly patients [22,23,24].

**Figure 2:**
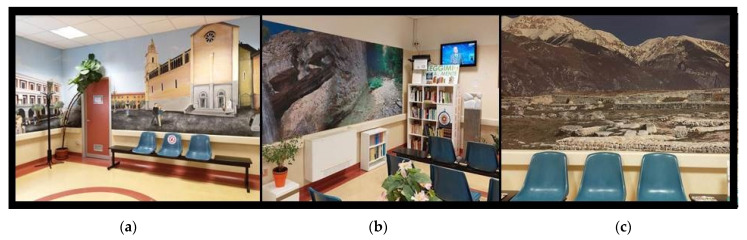
(**a**–**c**) Examples of environment’ “humanization” in Radiation Oncology Department of Chieti.

Furthermore, much attention has been given to the humanization processes of all department areas. Indeed, in recent years, great interest was dedicated to the importance of art and humanization in health environments, with particular regard to oncological departments, and several studies have shown the impacts of decorated care environments on perceptions of patients and on clinical outcomes [25]. A no-profit foundation, *IL Tratturo una Strada per la Vita*, in supporting the Chieti Radiation Oncology Center since 2009, has progressively decorated the walls and spaced in the department with images representative of Chieti city, e.g., photographic panels with naturalistic pictures about the Abruzzo region as well as a library, music and television in the waiting rooms (Figure 2a–c). This environment “humanization” process together with the staff attitudes could be related to the high and progressive score increases regarding welcome and comfort items.

The worst scores, on the other hand, were recorded for punctuality, reflecting a great expectation of respect for treatment schedules, as also reported in the benchmarks.

This RAMSI project mainly focused on two different periods: the dismissing of one LINAC and the COVID-19 period. After the obsolete LINAC decommission, better results were reported in all four areas, reflecting overall improvement. In particular, punctuality and professional skills increased (2% and 3%, respectively) due to a better adherence to appointments, the absence of treatment disruptions recorded with the obsolete LINAC and a greater use of hypofractionated schedules with less overall time of treatment fractions for patients.

During the COVID-19 period, an internal protocol for the prevention and management of patients, familiars, caregivers and health professionals was processed by the Chieti RT Department with hospital management. In national and international studies and guidelines [5,6,7,8,9,10,11,26,27,28], short treatments and home assistance, where possible, have been considered. Indeed, hypofractionated regimens were preferred in breast, prostate, oligometastatic and palliative treatments, and patient follow-up visits were conducted through telematic consultations. In comparing treatment activities prior to the full lockdown and after this period, no changes were observed in the numbers of services performed. Punctuality and professional skills scored the best results, whereas welcome and comfort were the worst because of the distress experienced by patients related toCOVID-19-safe management procedures. To the best of our knowledge, this is the first experience of applying a model for assessing the quality perceived by patients in radiotherapy in conditions of poor technological resources or in delicate periods such as the COVID pandemic.

The post-COVID-19 maintenance of hypofractionation schedules mainly in breast, prostate, oligometastatic and palliative cancers and the ongoing implementation of a second linear accelerator, which thus restored the ordinary equipment of the center, could improve these results. The experience is still ongoing and will continue in order to perform comparisons over time with the potential to expand the items, to include appointment booking experiences, staff empathetic to needs, doctor waiting times, and satisfaction with patients’ having a dedicated doctor.

## 5. Conclusions

A good radiotherapy service should improve medical outcomes, patients’ satisfaction and efficiency, making strong efforts to increase quality of treatment and service organization and reduce costs, becoming more patient-oriented and defining positive relationships with patients, familiars and caregivers. In this experience, the RAMSI project has provided a quick and easy evaluation methodology for assessing the perceptions of the radiotherapy service provided, especially in critical scenarios such as with limited equipment or during theCOVID-19 period. Moreover, this methodology led to corrective actions through a reorganization of work shifts and radiotherapy facilities. The RAMSI project is currently still evaluating radiotherapy performances and effective recognition of possible discomfort in patients’ care to enhance clinical procedures and the quality of treatments.

## Figures and Tables

**Table 1:** Overall collected feedbacks and related indexes during the observational period (February 2019–2022).

PREMs	Location	Total Feedbacks	Smile Index	Internal Benchmark	Smile-IN Approved
Welcome	Reception waiting room	869	8.4/10	8.8/10	86% (0–100%)
Comfort	Clinic waiting room	1267	8.9/10	8.6/10	92% (0–100%)
Professional skills	Treatment waiting room	1125	9.0/10	9.0/10	92% (0–100%)
Punctuality	Outside the LINAC’s bunker	2542	7.6/10	8.1/10	78% (0–100%)

**Table 2:** Data analysis related to the period before and after one LINAC decommissioning.

	FEB 2019-OCT 2020	NOV 2020-FEB 2022		FEB 2019-OCT 2020	NOV 2020-FEB 2022	
PREMs	Smile Index	Smile Index	Δ-Value	Smile-In Approved	Smile-In Approved	Δ-Value
Welcome	8.2/10	8.7/10	+0.5	85% (0–100%)	88% (0–100%)	+3%
Comfort	8.7/10	9.3/10	+0.6	90% (0–100%)	96% (0–100%)	+6%
Professional skills	8.8/10	9.1/10	+0.3	90% (0–100%)	93% (0–100%)	+3%
Punctuality	7.6/10	7.9/10	+0.3	77% (0–100%)	79% (0–100%)	+2%

**Table 3:** Data analysis related to the period before and after the COVID-19 lockdown.

	MAR 2019-MAY 2019	MAR 2020-MAY 2020		MAR 2019-MAY 2019	MAR 2020-MAY 2020	
PREMs	Smile Index	Smile Index	Δ-Value	Smile-In Approved	Smile-In Approved	Δ-Value
Welcome	8.4/10	7.5/10	−0.9	87% (0–100)	76% (0–100)	−9%
Comfort	8.7/10	8.4/10	−0.3	90% (0–100)	87% (0–100)	−3%
Professional skills	9.7/10	9.6/10	−0.1	97% (0–100)	96% (0–100)	−1%
Punctuality	8.2/10	8.1/10	−0.1	84% (0–100)	80% (0–100)	−4%

## Data Availability

The data presented in this study are available on request from the corresponding author.
